# “Fifty Shades” of Black and Red or How Carboxyl Groups Fine Tune Eumelanin and Pheomelanin Properties

**DOI:** 10.3390/ijms17050746

**Published:** 2016-05-17

**Authors:** Raffaella Micillo, Lucia Panzella, Kenzo Koike, Giuseppe Monfrecola, Alessandra Napolitano, Marco d’Ischia

**Affiliations:** 1 Department of Clinical Medicine and Surgery, University of Naples “Federico II”, Naples 80131, Italy; raffaella.micillo@unina.it (R.M.); monfreco@unina.it (G.M.); 2 Department of Chemical Sciences, University of Naples “Federico II”, Naples 80126, Italy; panzella@unina.it (L.P.); alesnapo@unina.it (A.N.); 3 Hair Care Products Research Laboratories, Kao Corporation, Tokyo 131-8501, Japan; koike.kenzo@kao.co.jp

**Keywords:** eumelanin, pheomelanin, melanins, 5,6-dihydroxyindoles, 5-*S*-cysteinyldopa, benzothiazines, dopachrome, melanocortin-1-receptor, antioxidant, pro-oxidant

## Abstract

Recent advances in the chemistry of melanins have begun to disclose a number of important structure-property-function relationships of crucial relevance to the biological role of human pigments, including skin (photo) protection and UV-susceptibility. Even slight variations in the monomer composition of black eumelanins and red pheomelanins have been shown to determine significant differences in light absorption, antioxidant, paramagnetic and redox behavior, particle morphology, surface properties, metal chelation and resistance to photo-oxidative wear-and-tear. These variations are primarily governed by the extent of decarboxylation at critical branching points of the eumelanin and pheomelanin pathways, namely the rearrangement of dopachrome to 5,6-dihydroxyindole (DHI) and 5,6-dihydroxyindole-2-carboxylic acid (DHICA), and the rearrangement of 5-*S*-cysteinyldopa *o*-quinoneimine to 1,4-benzothiazine (BTZ) and its 3-carboxylic acid (BTZCA). In eumelanins, the DHICA-to-DHI ratio markedly affects the overall antioxidant and paramagnetic properties of the resulting pigments. In particular, a higher content in DHICA decreases visible light absorption and paramagnetic response relative to DHI-based melanins, but markedly enhances antioxidant properties. In pheomelanins, likewise, BTZCA-related units, prevalently formed in the presence of zinc ions, appear to confer pronounced visible and ultraviolet A (UVA) absorption features, accounting for light-dependent reactive oxygen species (ROS) production, whereas non-carboxylated benzothiazine intermediates seem to be more effective in inducing ROS production by redox cycling mechanisms in the dark. The possible biological and functional significance of carboxyl retention in the eumelanin and pheomelanin pathways is discussed.

## 1. The Control Mechanisms of Carboxyl Groups in Melanogenesis

The dark insoluble eumelanins and the reddish-brown sulfur-containing pheomelanins are the primary determinants of visible pigmentation in man, mammals and birds and are both generated by a common enzymatic processes involving tyrosinase-catalyzed conversion of tyrosine to dopaquinone. The prevalence of either of these pathways is under genetic control and is biochemically determined by the non-enzymatic incorporation of cysteine accounting for a switch of the eumelanin pathway toward pheomelanin biosynthesis [1,2,3]. Traditionally, eumelanin has been regarded as the primary antioxidant and photoprotective agent in dark-skinned phenotypes [4,5]. Conversely, pheomelanin has been implicated as a major culprit in the enhanced susceptibility to UV-damage, melanoma and skin cancer of red-haired, fair-skinned individuals [6], due to its photosensitizing properties, leading to the production of high levels of reactive oxygen species (ROS) [7]. Although more than 120 genes are involved in regulation of cutaneous pigmentation, a key role seems to be played by the *MC1R* gene encoding for melanocortin-1-receptor (MC1R), a 317-amino acid G protein-coupled receptor expressed prevalently on the surface of melanocytes. The *MC1R* gene is regulated positively by α-melanocyte-stimulating hormone (α-MSH) and negatively by antagonists of MC1R, such as Agouti signal protein (ASIP) and beta-defensin 3, competing with α-MSH [8,9].

In wild-type eumelanic subjects, α-MSH-mediated stimulation of *MC1R* results in activation of the associated Gs protein, which activates adenyl cyclase and increases the levels of cellular cyclic adenosine 3’,5’-monophosphate (cAMP), which is responsible for many of the effects of α-MSH, such as activation of tyrosinase (the rate-limiting enzyme in melanogenesis), tyrosinase-related protein 1 (Tyrp1) and dopachrome tautomerase (Dct, also called tyrosinase-related protein 2), to produce eumelanins [10,11].

**Scheme 1:**
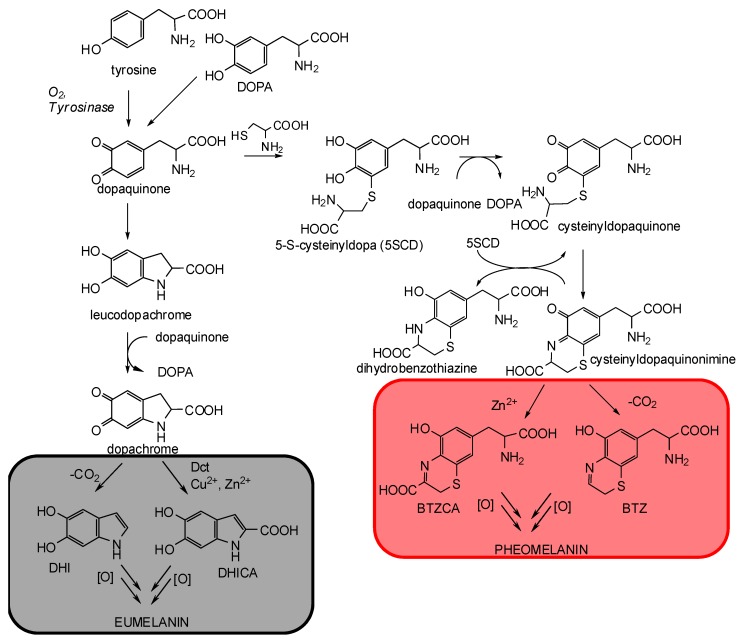
Schematic view of eumelanin and pheomelanin biosynthetic pathways.

Eumelanin synthesis starts from tyrosinase-catalyzed oxidation of tyrosine to dopaquinone (Scheme 1) [12,13,14]. Dopaquinone then undergoes intramolecular addition of the amino group onto the *o*-quinone moiety giving cyclo-DOPA (or leucodopachrome). Redox exchange between leucodopachrome and dopaquinone then leads to the formation of dopachrome, which, *in vitro*, undergoes spontaneous decarboxylation giving rise to 5,6-dihydroxyindole (DHI). *In vivo*, however, the tyrosinase-related protein Dct directs the fate of dopachrome toward tautomerization with non-decarboxylative formation of 5,6-dihydroxyindole-2-carboxylic acid (DHICA), a major circulating melanogen [15]. Thus, while natural eumelanins contain more than 50% of DHICA-derived units, synthetic DOPA melanin consists for the most part of DHI with some 10% DHICA [14]. Interestingly, in addition to Dct, also a number of biorelevant transition metal ions, such as Cu^2+^ and Zn^2+^, can direct the rearrangement of dopachrome to DHICA at physiological pH [16,17,18].

Specific mutations at the *MC1R* gene are involved in the switch from eumelanogenesis to pheomelanogenesis [19]. Variants or polymorphism of *MC1R* may result in loss of receptor functions associated with α-MSH binding or cAMP signaling. Among red-haired individuals, homozygous for alleles of the *MC1R* gene, different degrees of decreased function can be found. These are associated with a failure in tyrosinase activity favoring the intervention of cysteine in the melanogenesis pathway [19]. As a result, the formation of pheomelanin pigments is predominant over that of eumelanins. Non-enzymatic addition of the SH group of cysteine to dopaquinone leads to 5-*S*-cysteinyldopa (5SCD) and minor amounts of 2-*S*-cysteinyldopa (2SCD), in a ratio of about 5:1 [1,20]. Oxidation of cysteinyldopas followed by intramolecular cyclization then leads to a transient *o*-quinoneimine, which can either react with the parent cysteinyldopa to give a dihydrobenzothiazine by redox exchange or isomerize with or without decarboxylation to give the 2*H*-1,4-benzothiazine (BTZ) or its 3-carboxylic acid (BTZCA) derivatives (Scheme 1) [21,22]. Notably, in the presence of Zn^2+^ ions, one of the most abundant trace element present in skin and hair, decarboxylation of the quinoneimine is substantially inhibited, and BTZCA accumulates in the reaction medium as the main product due to the stabilizing effect of the metal ion [23,24].

## 2. The Impact of Carboxylated Units on Eumelanin Structure and Properties

5,6-Dihydroxyindoles are easily oxidized to produce black or dark brown insoluble pigments via complex mixtures of products. Several oligomer intermediates in the oxidation of DHI and DHICA have been isolated and characterized.

**Figure 1:**
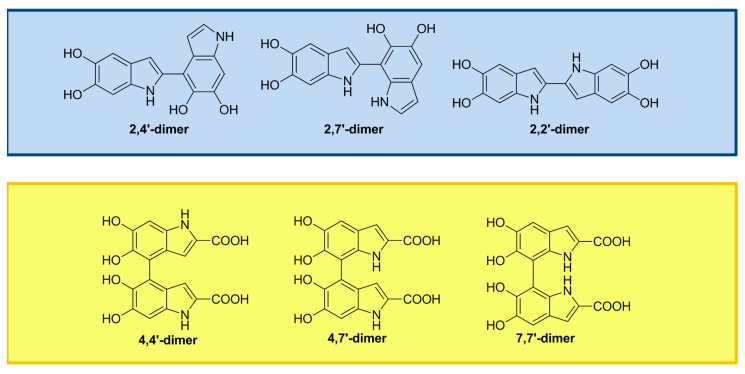
Main oxidation products of DHI (blue box) and DHICA (yellow box).

DHI oxidation gives rise to dimers, trimers and tetramers, primary coupled through 2,2’-, 2,4’- and 2,7’-bonding (Figure 1) [25,26,27,28,29].

DHICA polymerization is conditioned by the presence of the carboxylic acid group at the 2-position, which decreases nucleophilicity on the pyrrole moiety through its electron-withdrawing nature, thereby directing reactivity mainly towards the 4,4’-, 4,7’- and 7,7’-bonding formation, with lower involvement of the 3-position (Figure 1) [25,26,30].

**Figure 2:**
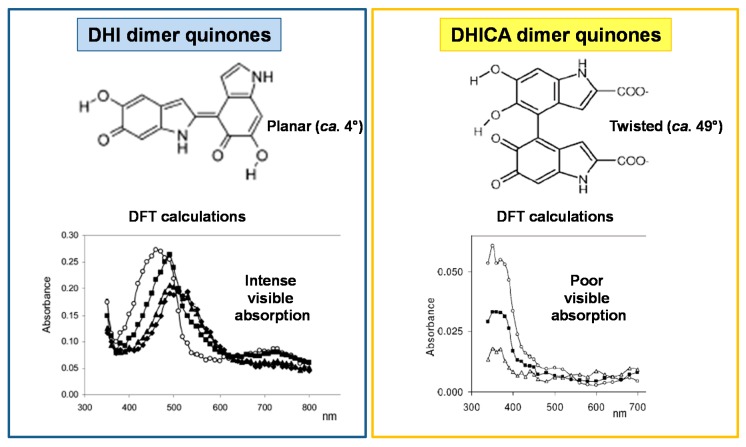
Predicted structures and experimental UV-visible absorption spectra at various times of the oxidation mixtures of DHI and DHICA oligomers. Blue box: change in absorption at various times after pulse radiolysis of a N_2_O-satured aqueous solution of DHI dimer (1.5 × 10^−4^ M) in 0.5 M KBr/7.0 × 10^−2^ M phosphate buffer, pH 7.0: (○) 14 μs; (■) 102 μs; (▲) 402 μs; (♦) 866 μs. Yellow box: changes in absorption at various times after pulse radiolysis of DHICA dimer (1.5 × 10^−4^ M) in 0.5 M KBr/7.0 × 10^−2^ phosphate buffer, pH 7.0: (○) 400 μs; (■) 5 μs; (Δ) 44 ms [31,32].

Oligomers of DHICA exhibit structural features different from those of DHI. While the latter oligomers can adopt planar conformations, DHICA polymerizes mainly through biphenyl-type bonds, resulting in the generation of non-planar, partly linear backbones exhibiting hindered rotation (atropisomerism) at the interunit bonds [31,32]. Deviation from coplanarity in DHICA oligomers is supported by the negative charge of the carboxylate groups forcing twisting about the inter-ring bond. The so-formed twisted backbones cannot give rise to π-stacked supramolecular aggregates, at variance with the largely planar oligomeric scaffolds derived from DHI. Therefore, while DHI dimers generate on oxidation largely planar species absorbing strongly in the visible range, DHICA oligomers do not produce significant visible absorption above 400 nm, due to inter-unit dihedral angles of *ca*. 49° with localized *o*-quinone moieties and significant interruption of inter-unit π-electron delocalization (Figure 2) [33,34].

**Figure 3:**
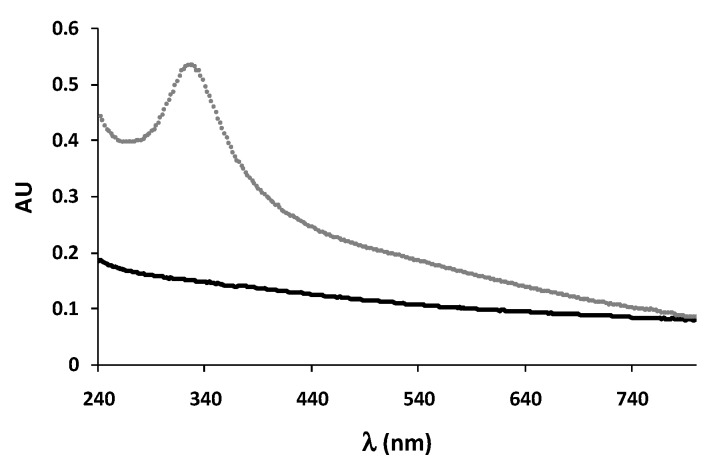
UV-visible spectra of DHI (black trace) and DHICA melanin (gray trace) at pH 7.5.

Figure 3 shows the absorption spectra of DHI and DHICA melanins at pH 7.5. While DHI melanin gives a nearly monotonic profile [35], the DHICA polymer displays an intense absorption band in the UV region around 320 nm [36]. This latter feature is suggestive of the presence of reduced monomer-like chromophoric components co-existing with quinonoid units and persisting during the polymerization process as a consequence of the hindered inter-unit π-electron delocalization within oligomer/polymer scaffolds. Consistent with this view, treatment of DHICA melanin with a reducing agent such as NaBH_4_ did not affect the 320-nm band, but induced a decrease of the absorption in the visible region, suggesting the contribution in the latter of reducible quinonoid chromophores [36,37].

**Figure 4:**
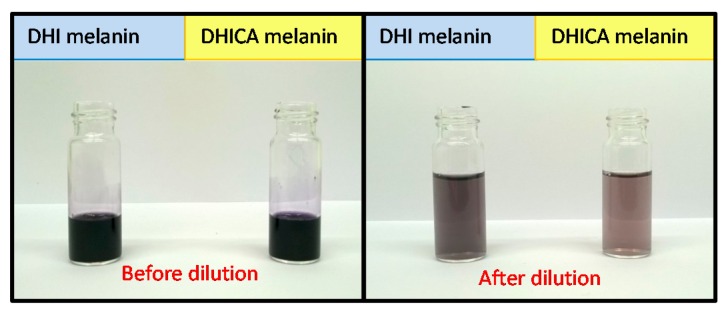
Melanins from DHI (blue box) and DHICA (yellow box) oxidation before and after 1:90 dilution in 1% PVA-containing buffer.

Recently, a comparison of the UV-visible spectra of soluble melanins from DHI and DHICA in the presence of polyvinyl alcohol (PVA) was carried out [38,39]. Despite apparent similarities, the absorption spectra of the two pigments differed in the presence of a pronounced broad band around 500 nm in the case of DHI-derived eumelanin, which was negligible for the DHICA polymer. Notably, upon dilution in PVA-containing buffer, DHICA-melanin gradually lost its visible absorption properties (the “black” color), whereas DHI-melanin did not (Figure 4).

**Figure 5:**
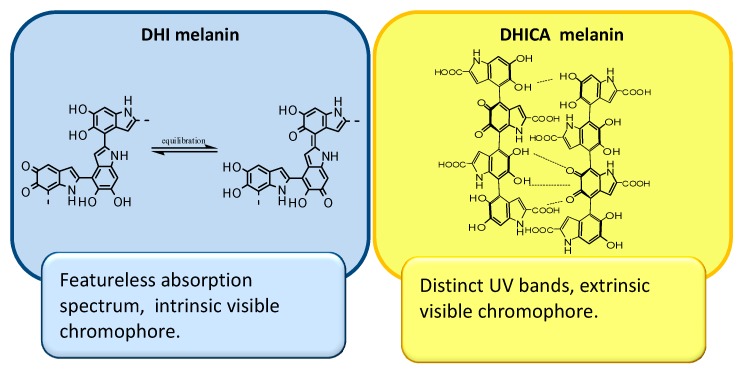
Intrinsic and extrinsic contributions to the chromophoric properties of DHI and DHICA melanin.

Consequently, it has been suggested that while the DHI-melanin chromophore is determined for the most part by intrinsic effects relating to efficient π-electron delocalization within the largely planar oligomeric scaffolds, the black chromophore of DHICA melanin derives largely from aggregation-dependent intermolecular perturbations of the π-electron systems, being therefore mainly extrinsic in character (Figure 5) [38].

The presence of unpaired electrons in melanin is one of the key features of the pigment that is involved in its antioxidant properties [40,41,42]. Electron paramagnetic resonance (EPR) spectra of DHI and DHICA melanins showed very similar *g*-values, but a significantly narrower signal was observed for the DHICA melanin [36]. A remarkable difference was also apparent from the power saturation curves, which concurred with the suggestion of a lower dispersion and a greater homogeneity of the free radical components in the DHICA melanin. Both data would suggest the presence of relatively homogeneous free radical species in DHICA melanin, spatially confined within restricted segments of the polymers, in contrast with the broader variety of free radical species that could be generated within the delocalized π-electron systems of the DHI polymer. In addition, the free radical population of DHICA melanin is markedly affected by intermolecular interactions, as a further decrease in the ΔB value was determined for DHICA melanin in the presence of 1% PVA hindering aggregation, whereas no differences were observed in the case of the pigment from DHI [36].

Dct inactivation in knockout mice, supposedly associated with a decrease in the DHICA content of melanin, has been reported to elevate the level of ROS following exposure to UVA radiation and to increase the number of sunburned and apoptotic cells in the epidermis [43]. DHICA-melanin was also found to be a much more potent hydroxyl radical-scavenger *in vitro* compared to DHI melanin, suggesting that Dct regulates eumelanin antioxidant properties. The superior antioxidant and free radical scavenger properties of DHICA melanin relative to DHI melanin were confirmed in three different tests, *i.e.*, the 1,1-diphenyl-2-picrylhydrazyl (DPPH), 2,2′-azinobis(3-ethylbenzothiazoline-6-sulfonic acid) (ABTS) and nitric oxide (NO) scavenging assays (Table 1) [36].

**Figure 6:**
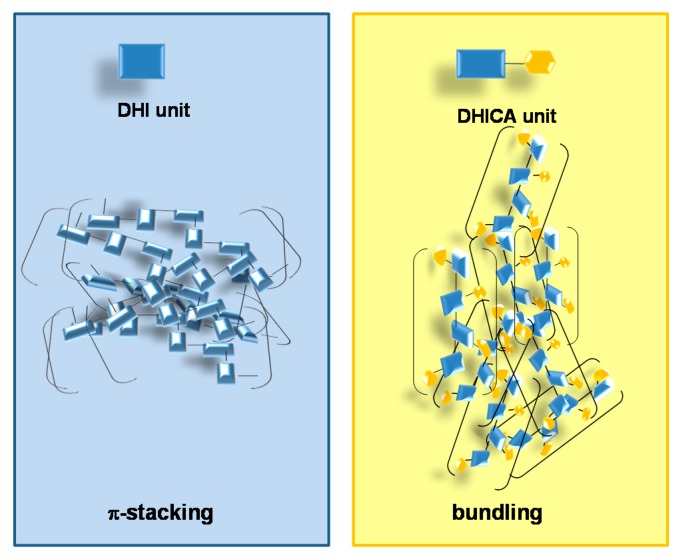
Simplified representation of aggregates’ formation for DHI and DHICA melanins.

This difference was ascribed to the de-stabilizing effects of non-planar structures on electron delocalization and aggregation, imparting monomer-like behavior to the polymer. Formation of weak aggregates would then account for a greater accessibility of free radicals compared to the case of compact π-stacked DHI melanin (Figure 6).

## 3. The Impact of Carboxylated Units on Pheomelanin Structure and Properties

**Figure 7:**
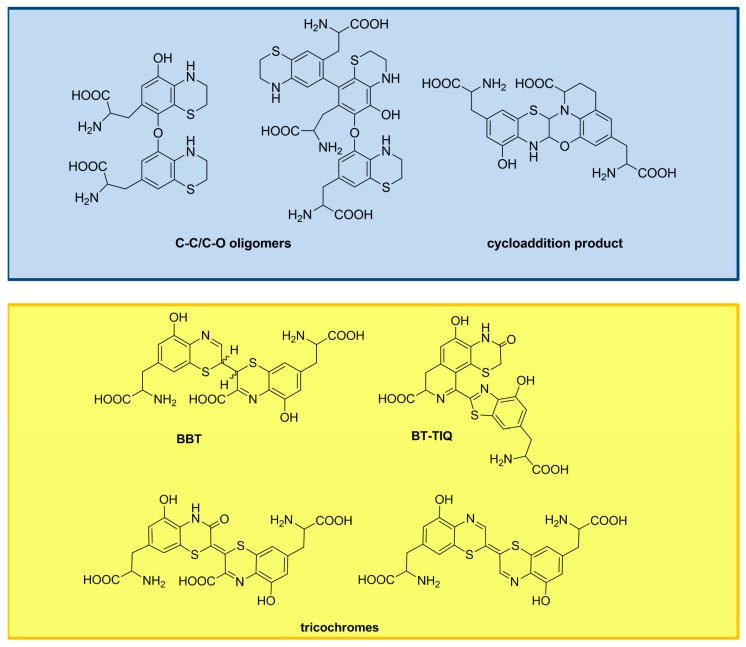
Main oxidation products of BTZ (blue box) and BTZCA (yellow box).

Pheomelanin pigments are formed by oxidative polymerization of BTZ and BTZCA, which may follow different paths depending on the presence of the carboxyl group on the 3-position. Oligomers characterized by C–C and C–O bonds were isolated from the peroxidase/H_2_O_2_ oxidation mixture of 5SCD via BTZ after reductive treatment (Figure 7) [44], whereas an unusual cycloaddition product was formed during the tyrosinase-catalyzed oxidation (Figure 7) [45]. In contrast, oxidation of BTZCA leads to the 2,2’-bibenzothiazines (BBT) [23]. Further oxidative steps would lead to the trichochromes [46]. A main dimeric product of BTZCA, featuring isoquinoline and benzothiazole moieties (BT-TIQ), has been characterized as the most important building block of natural pigments from red hair and chicken feathers (Figure 7) [47,48,49].

Apart from conferring a weak protecting capacity against UV radiation, pheomelanins are known to act as potent photosensitizers leading to ROS production [3,7,50,51,52,53,54,55,56,57]. Indeed, pheomelanosomes from red human hair exhibited a lower ionization potential with respect to eumelanosomes, with a photoionization threshold falling in the UVA region of the spectrum, at *ca*. 326 nm, in the case of pheomelanin, and in the UVB region, at *ca*. 280 nm, in the case of eumelanins [58].

Excitation of the pheomelanin chromophore to the singlet state followed by decay to the triplet state with transfer of an electron to oxygen and the formation of the superoxide anion was suggested in early studies [50,59]. This latter would indirectly induce DNA oxidative damage [55] through its downstream products, such as hydrogen peroxide and hydroxyl radicals. Transfer of excitation to oxygen would lead to singlet oxygen (^1^O_2_) capable of destruction of the pheomelanin chromophore [60] accounting for the low absorbance, both in the visible and UV range, of UVA-irradiated pheomelanin samples. This would explain the poor photoprotective capacity of skin in the red-haired phenotype [61].

Pump-probe spectroscopic measurements suggested carboxylated benzothiazine units like those present in BBT, with absorption maxima around 350–360 nm, as the reactive chromophore involved in pheomelanin photoreactivity [62,63]. Furthermore trichochromes were shown to access a long-lived excited state upon visible light excitation [64]. Interestingly, model pheomelanins produced in the presence of zinc ions and, hence, expectedly richer in BTZCA-derived structural units exhibited enhanced oxygen consumption rates (Table 2) and produced higher fluxes of superoxide following irradiation with UVA and visible light [52], pointing to BT-TIQ, BBT and other related units as the main determinants of pheomelanin phototoxicity.

**Figure 8:**
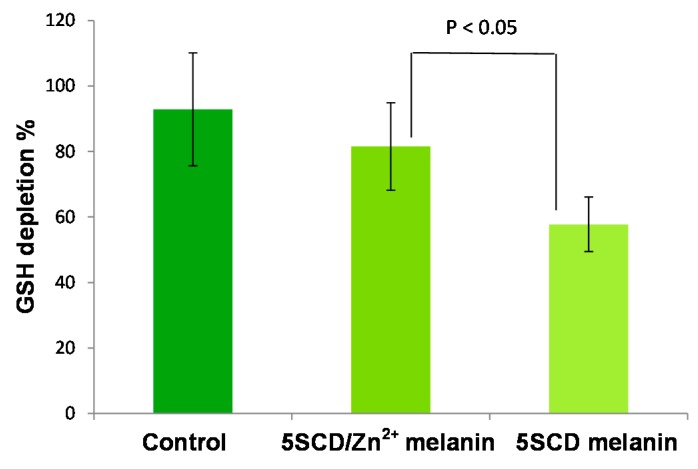
Glutathione (GSH) depletion (%) after 3 h in 0.1 M phosphate buffer (pH 7.4) in the absence or in the presence of 5SCD melanin prepared in the absence or in the presence of Zn^2+^ ions. Reported are the mean ± SD values from at least five experiments. Significant differences were determined by an independent sample two-tailed *t*-test.

Pheomelanins have also been implicated in UV-radiation-independent carcinogenic contributions to melanomagenesis by inducing oxidative DNA damage [65,66,67]. The ability of pheomelanin to promote antioxidant depletion has been reported in a recent paper [68] showing that the natural pigment isolated from red hair [69] is able to markedly accelerate the autoxidation of two key cellular antioxidants, *i.e.*, glutathione (GSH) and NAD(P)H. This reaction took place in the presence of oxygen and has been ascribed to the property of pheomelanin pigment to act as a redox cycling agent accepting H-atoms from the substrates and transferring electrons to oxygen [70]. Notably, this pro-oxidant activity was found to be lower for synthetic pigments prepared in the presence of zinc ions compared to that produced in the absence of the metal (Figure 8), which would point to BTZ-derived intermediates as the active structural units in ROS production by pheomelanin in the dark [71].

## 4. Conclusions

Retention of carboxyl groups in eumelanins and pheomelanins may be under both enzymatic and non-enzymatic control during melanogenesis and appears to play a fundamental role in controlling the structural, light-absorbing and free radical properties of the pigments. As summarized in Table 3, synthetic model pigments with a higher proportion of carboxylated units exhibit peculiar physicochemical properties, which suggest a specific biological role and justify the operation of more or less sophisticated control mechanisms.

The explanation for these differences may be sought for in the peculiar features of carboxylated units, including: (1) a lower number of reactive sites available for polymerization and cross-linking; (2) a lower oxidation potential; and especially; (3) a negative charge profoundly affecting the structural properties of oligomers and their susceptibility to post-synthetic modifications.

While in the case of pheomelanin, the role of carboxyl groups is only partially elucidated, their impact on eumelanin properties is now established and explains a major *conundrum* in pigment cell biology: why have an enzymatically-controlled pathway to produce a poor pigment precursor like DHICA instead of exploiting the spontaneous, biologically “low cost” process for DHI formation, giving rise to dark compact and intensely-colored pigments. Although caution must be exercised before extending conclusions from *in vitro* studies to the *in vivo* situation, this latter point may be taken as an additional indirect argument against a merely pigmentary role of melanins in nature.

## Figures and Tables

**Table 1:** Free radical scavenging properties of DHICA and DHI melanins [36].

Melanin	DPPH (%)	ABTS (%)	NO (%)
DHICA melanin	100 ± 4	46 ± 2	64 ± 3
DHI melanin	29 ± 1	4 ± 1	26 ± 1

**Table 2:** Oxygen consumption rate by 5SCD melanins prepared in the presence or in the absence of Zn^2+^ ions upon irradiation with UVA or blue light [52].

Melanin	UVA-Induced Oxygen Consumption Rate (mM/min)	Blue Light-Induced Oxygen Consumption Rate (mM/min)
5SCD/Zn^2+^ melanin	0.12 ± 0.02	0.13 ± 0.02
5SCD melanin	0.032 ± 0.005	0.024 ± 0.004

**Table 3:** Comparison of the properties of eu-/pheo-melanins from carboxylated/non-carboxylated precursors.

Monomer Characteristics	DHI	DHICA (Carboxylated)	BTZ	BTZCA (Carboxylated)
Reactive sites	At least 4	Usually 2	At least 3	Usually 1
Oxidizability	High	Low	High	Low
**Polymer properties**				
Solubility	Nihil	Very slight ^1^	Low ^1^	Moderate ^1^
EPR signal intensity	Strong	Weak	Weak	Intense
Visible absorption	Intense	Poor	Moderate	Intense
Photophysical properties	Efficient excited state decay	Highly efficient excited state decay	Poor photosensitizer	Strong photosensitizer
Chemical properties	Antioxidant	Highly potent antioxidant	Strong pro-oxidant	Weak pro-oxidant

^1^ at neutral pH.

## Abbreviations

DHI	5,6-dihydroxyindole
DHICA	5,6-dihydroxyindole-2-carboxylic acid
BTZ	1,4-benzothiazine
BTZCA	1,4-benzothiazine carboxylic acid
UVA	ultraviolet A
ROS	reactive oxygen species
MC1R	melanocortin-1-receptor
α-MSH	α-melanocyte stimulating hormone
ASIP	Agouti signal protein
cAMP	cyclic adenosine 3’,5’-monophosphate
Tyrp1	tyrosinase-related protein 1
Dct	dopachrome tautomerase
5SCD	5-*S*-cysteinyldopa
2SCD	2-*S*-cysteinyldopa
PVA	polyvinyl alcohol
EPR	electron paramagnetic resonance
DPPH	1,1-diphenyl-2-picrylhydrazyl
ABTS	2,2′-azinobis(3-ethylbenzothiazoline-6-sulfonic acid)
NO	nitric oxide
BBT	2,2’-bibenzothiazines
BT-TIQ	benzothiazolylthiazinodihydroisoquinoline
GSH	glutathione
